# Adult attachment style and maternal-infant bonding: the indirect path of parenting stress

**DOI:** 10.1186/s40359-020-00424-2

**Published:** 2020-06-08

**Authors:** Dag Nordahl, Kamilla Rognmo, Agnes Bohne, Inger Pauline Landsem, Vibeke Moe, Catharina Elisabeth Arfwedson Wang, Ragnhild Sørensen Høifødt

**Affiliations:** 1 Department of Psychology, Faculty of Health Sciences, UiT The Arctic University of Norway, Tromsø, Norway; 2 Division of Child and Adolescent Health, University Hospital of Northern Norway, Tromsø, Norway; 3 Department of Health and Care Sciences, Faculty of Health Sciences, UiT The Arctic University of Norway, Tromsø, Norway; 4 Department of Psychology, Faculty of Social Sciences, University of Oslo, Oslo, Norway; 5 Regional Centre for Child and Adolescent Mental Health, East and South, Norway

**Keywords:** Parenting stress, Attachment style, Bonding, Maternal-infant bonding, Mother-infant relationship, Mediation

## Abstract

**Background:**

The quality of maternal-infant bonding is related to important child outcomes. The literature has assumed that the ability to form relationships is a relatively stable trait, and research studies have suggested that a mother’s attachment style in close adult relationships is related to mother-infant bonding. The transition to parenthood is also often stressful, and the adult attachment style may relate to parenting stress in the first year after birth. Such stress could possibly have a negative relationship with the mother-infant bond. In the present study, we examined the associations between maternal adult attachment styles and the quality of mother-infant bonding and whether this relationship is mediated by parenting stress.

**Methods:**

The present study sample comprised 168 women (mean age 31.0 years, SD 4.23 years). Between weeks 31 and 41 of gestation, the anxious and avoidant adult attachment dimensions were measured with the Experiences in Close Relationships questionnaire (ECR). Between 5 and 15 weeks after birth mother-infant bonding and parenting stress were measured with the Maternal Postnatal Attachment Scale (MPAS) and the Parenting Stress Index-Parent Domain (PSI-PD), respectively.

**Results:**

Both attachment-related avoidance and attachment-related anxiety correlated significantly and negatively with mother-infant bonding. However, a regression analysis showed that only attachment-related avoidance was a significant predictor of mother-infant bonding when controlling for demographic variables and maternal mental health history. The relationship between the adult attachment style and bonding was mediated by parenting stress. Higher scores on attachment avoidance and anxiety were related to increased stress, which was related to decreased quality of bonding. The overall parent domain and the subscale of competence in the parent-related stress dimension mediated between attachment avoidance and bonding, and the overall parent domain and the subscales of competence and role restriction mediated between attachment anxiety and bonding. There was no direct relationship between the adult attachment style and mother-infant bonding when parenting stress was included as a mediator.

**Conclusions:**

This study illustrates that maternal adult attachment style relates to mother-infant bonding. This relationship was mediated by parenting stress. The results may have implications for the early identification of mothers at risk of having bonding difficulties.

## Background

Maternal bonding, defined as an emotional tie [1] from the mother towards her child, develops already during pregnancy [2–4]. The quality of maternal-infant bonding is important, as it is predictive of maternal sensitivity [5] and is related to child developmental outcomes [6, 7]. Thus, research on factors related to the quality of mother-infant bonding is important.

Bonding is related to the concept of attachment. It has been argued that while attachment encompasses care seeking, bonding might be more related to caregiving [8]. Thus, these two concepts might tap into different aspects of the parent-child relationship. Attachment theory states that establishing close bonds/relations to others is of central importance to all humans, irrespective of age [9]. This is further elaborated with the construct of internal working models, which are assumed to develop during early childhood from experiences with important caregivers, and to have an impact throughout life on the way we relate to ourselves and other people [10]. Hence, it is reasonable to assume that a mother’s attachment style will affect her bonding with the infant. Additionally, adults are theorized to form attachments to each other, and adult attachment styles are often defined by feelings and behaviors in close relationships, often romantic relationships, during adulthood [10]. Adult attachment can be conceptualized as a two-dimensional model of attachment-related anxiety and attachment-related avoidance (described in [11, 12]). Attachment anxiety is about, e.g., fear of not being loved by a partner or that a partner may leave you. Attachment avoidance concerns unease with dependency and intimacy [12, 13].

Adult attachment styles have been studied in relation to diverse aspects of parenting (for a review see: [14]), and the relationship between adult attachment styles and maternal bonding has received some attention [15–18]. Van Bussel, Spitz and Demuttenaere [15] found weak associations between adult attachment patterns and maternal bonding. Chrzan-Detkos and Lockiewics [16] found a relationship between adult attachment and maternal bonding during pregnancy, but not with maternal bonding after birth. However, two studies [17, 18] found a relationship between adult attachment style and maternal bonding postpartum, and this relationship was mediated by depression. The variability in the results of the studies investigating the association between adult attachment style and maternal bonding makes evident the need for more research on this relationship and on the potential mediators of this link.

The transition to parenthood is considered stressful [19], inducing changes in many areas of life. Such stress may impact the mother-infant relationship [20, 21]. Parenting stress typically occurs when parenting demands exceed the parents’ sense of accessible resources [22]. Parenting stress is often thought to arise from perceived challenges with the child’s behavior, the relationship between the parent and child, and aspects related to the parent’s health, life situation and perception of the parental role [22]. There is support for a negative relationship between parenting stress and maternal-infant bonding [6, 23]. Parenting stress is suggested related with decreased sensitivity in parents, and increased intrusivesness and hostility towards the child [24]. Individuals with difficulties in close relationships due to insecure attachment styles may experience increased stress as parents, feeling frustrated and overwhelmed, which may spill over to the quality of the mother-infant bond. Maternal-infant bonding is also negatively affected by mothers’ depressive symptoms during pregnancy [3] and after birth [4, 25–27], depressive symptoms in both spouses [25], and symptoms of posttraumatic stress disorder [28].

As with maternal-infant bonding, the relationship between adult attachment and parenting stress has also been investigated in some studies. Adult attachment style has been found to relate to parenting stress, measured in the first year after birth [29–32]. Studies have found that attachment anxiety and attachment avoidance is related to increased parenting stress [14]. A recent study indicates that this effect is most prominent in mothers with high level on the attachment anxiety dimension. This includes mothers’ sense of competence in parenting, partner support, isolation and the degree of feeling restricted by the parenting role [33]. The relationship between attachment style and parenting stress may be understood within attachment theory. In this theory, internal working models are thought to influence the perception of and coping with stressful experiences [34]. Thus, people with secure or insecure attachment patterns may have different ways of experiencing and coping with stressful events [34]. Hence, individuals with anxious or avoidant attachment styles may experience more relational distress and may also tend to cope with this distress in non-optimal ways [35]. This group may therefore be more prone to experiencing higher levels of parenting stress.

### Study aims

Mothers’ adult attachment styles, parenting stress and maternal-infant bonding seem to be related. Parenting stress in the first year after birth has been related both to the parent’s adult attachment style [29–32] and to maternal-infant bonding [6, 23]. The aim of the present study is twofold. First, we will explore associations between mothers’ adult attachment styles and their bonding with their infants. Second, we will investigate parenting stress as a potential mediator between adult attachment style and maternal bonding postpartum. Parenting stress will be explored as a domain, but with a main focus on the subscales.

## Method

### Participants and procedure

The present study is part of the Northern Babies Longitudinal Study (NorBaby) [36] on risk factors of parental mental health problems, poor parent-infant interaction and infant development. Midwives recruited Norwegian speaking participants from the municipality of Tromsø during pregnancy. One week later, a member of the research team called the participants and planned a meeting for enrollment in the study. Between October 2015 and December 2017, 220 women and 130 partners were included in the study. From pregnancy to 6 months postpartum participants completed 6 measurement points: T1–T3 during pregnancy and T4–T6 after birth.

For the present study, only female participants who completed questionnaires relevant for the present study at T1 (between gestational weeks 13 and 34, mean 23.0, SD 3.61, median 23.0), T3 (between gestational weeks 31 and 41, mean 34.3, SD 2.18, median 34.0) and T4 (between 5 and 15 weeks after birth, mean 8.0, SD 1.74, median 8.0) were included. The reason for the time span for each measurement point are recruitment at different weeks of gestation (impacting responses at T1 and T3), and late responses from the participants (impacting responses at T3 and T4). Data were collected using an Internet-based survey during a meeting with a member of the research team (T1) and at home (T3 and T4).

Within 4 weeks of postdelivery, a subset of the families in the study (*n* = 92) completed up to three sessions with the Newborn Behavioral Observation program (NBO: [37]). In the present study sample, 71 (42.3%) of the participants were in the group participating in the NBO. The NBO is assumed to have the potential to enhance the relationship between the parent and infant, although it showed few effects on parenting stress and bonding in an earlier study based on the NorBaby study sample [38]. An investigation of the effects of the NBO is not part of the present study, but group membership will serve as a control variable.

### Measures

#### Demographic and health information

Demographic information was collected at T1 and included maternal age, education, gross annual household income, marital status, whether the pregnancy was wanted and whether this was the parents’ first child. Further, participants answered three questions concerning their mental health: first, whether they have been in contact with professionals for mental health issues; second, whether they have previously experienced being depressed most of the day and almost every day for a period of 2 weeks; third, whether they have previously had a two-week period of diminished ability to enjoy the things that they usually find enjoyable. In addition, the rate of premature births was calculated by determining the difference between the mothers’ reported due date and the actual day of birth. Premature birth was defined as being born more than 3 weeks before the due date.

#### Adult attachment style

Adult attachment style was measured with the Experiences in Close Relationships questionnaire (ECR: 12) at T3. The ECR consists of 36 items, all of which are answered on a 7-point scale, from strongly disagree (1) to strongly agree (7). The 36 items are divided into the two dimensions of avoidance (18 items) and anxiety (18 items). The ECR mainly addresses attachment in romantic relationships. For example, the avoidance scale includes a focus on the degree of discomfort with being close to a romantic partner and the anxiety scale measures, among other things, the participants’ worries about being abandoned and not being close enough to romantic partners. In the present study, Cronbach’s alpha was 0.90 for ECR avoidance and 0.91 for ECR anxiety.

#### Parenting stress

Parenting stress was measured with the Parenting Stress Index – Parent Domain (PSI-PD: [39]) at T4. The PSI-PD is part of the PSI and is designed to measure stress in the parenting role and the relationship between parent and child. The PSI-PD consists of 7 subscales: competence in the parenting role (PSI-CO; 13 items), social isolation (PSI-IS; 6 items), support from spouse (PSI-SP; 7 items), depression (PSI-DP; 9 items), role restriction by the parenting role (PSI-RO; 7 items), attachment (PSI-AT; 7 items), and health (PSI-HE; 5 items). All items are answered using 4 or 5 response options. For the present study, the PSI-PD, a shortened version of the PSI-PD (PSI-PD5) and 5 of the subscales were used (PSI-CO, PSI-IS, PSI-SP, PSI-DP, and PSI-RO). The PSI-HE subscale was not considered theoretically relevant, and the PSI-AT subscale had a too close a thematic resemblance to the outcome measure. For the PSI-PD5, we therefore removed PSI-AT and PSI-HE from the composite score and did not include them when we tested the subscales as individual mediators. The Norwegian version of PSI has been translated by J. A. Rønning and has been used in earlier research [40]. In the present sample, Cronbach’s alpha was 0.91 for PSI-PD, 0.90 for PSI-PD5, 0.77 for PSI-CO, 0.73 for PSI-IS, 0.64 for PSI-SP, 0.81 for PSI-DP and 0.71 for PSI-RO.

#### Parental bonding

Maternal bonding with the infant was measured with the Maternal Postnatal Attachment Scale (MPAS: [41]) at T4. The MPAS measures the emotional quality of bonding, hostility towards the child and the degree of pleasure in interacting with the child. The scale consists of 19 items. The items have varying response options, ranging from 2 to 5 response options. Eight items were reversed, and all response options were recoded, such that a score of 1 represented low bonding and 5 represented high bonding. The MPAS was translated to Norwegian by members of the research team in consultation with a professional translator. In the current study, Cronbach’s alpha was 0.81 for the MPAS.

### Statistical analysis

There were indications of challenges with normality, as skewness and/or kurtosis were above 1 for the MPAS, PSI-DP and ECR avoidance. Hence, a nonparametric approach was applied with Spearman’s correlations and bootstrapping generated confidence intervals using 10,000 samples for the hierarchical regression analysis and mediation analyses. A hierarchical regression approach was applied to explore the effects of eight confounders (see Table 1) and the two ECR dimensions of anxiety and avoidance on the MPAS. In the first block, we included ECR anxiety and avoidance as predictors. In the second block, demographic information (maternal age, parenting experience, education, and family income) and allocation to the NBO or the control group were added. The mental health variables of mental health help seeking, previous experience with depression and previous lack of joy were added in the third block. Whether the pregnancy was wanted, marital status and prematurity were excluded due to little variability in the scores. The variables of gross annual household income and education were dummy coded, and parenting experience was recoded as “first-time parent” and “one or more previous children”. A mediation analysis was conducted to investigate the indirect effect of ECR avoidance and anxiety on the MPAS through the PSI and to investigate their direct effect on the MPAS, while controlling for the PSI. First, we conducted mediation analyses with PSI-PD as a mediator. Second, we removed the subscales PSI-AT and PSI-HE from the PSI-PD and conducted mediation analyses with a composite of the remaining five subscales (PSI-PD5) as a mediator. Third, we conducted mediation analyses with the five subscales PSI-CO, PSI-IS, PSI-SP, PSI-DP, and PSI-RO as mediators. The eight potentially confounding variables used in the hierarchical regression analysis were all included as covariates in the mediation analyses. SPSS version 25 was used for the hierarchical regression analysis and descriptive statistics, and PROCESS version 3.1 [42] was used for the mediation analyses.

**Table 1:** Sample demographics

Characteristics at T1	Mean (SD)	N (%)
Maternal age (years)	31.0 (4.23)	
Pregnancy wanted^a^		
Yes		161 (95.8)
Do not know		3 (1.8)
Parenting experience		
First-time mother		83 (49.4)
Second-time mother		67 (39.9)
Two or more previous children		18 (10.7)
Maternal education		
Upper secondary school or lower		20 (11.9)
Up to 4 years higher education		51 (30.4)
4 or more years higher education		97 (57.7)
Gross annual household income^b^		
350,000 NOK (39,672 USD) or less		7 (4.2)
351,000–750,000 NOK (39,785–85,011 USD)		41 (24.4)
751,000 NOK (85,125 USD) or more		119 (70.8)
Marital status		
Married or cohabiting		166 (98.8)
Single		2 (1.2)
Maternal mental health history		
Have been in contact with professionals for mental health issues		51 (30.4)
Previous experience with being depressed most of the day, almost every day for a period of two weeks		55 (32.7)
Having previously had a two-week period of diminished ability to enjoy things one has usually found enjoyably		68 (40.5)
Premature birth (more than 3 weeks before due date)^a^		6 (3.6)

*N* = 164–168; ^a^Four missing values; ^b^One missing value

### Missing values

Data from one participant were not included due to completion of T3 after birth. In addition, one participant withdrew from the study before completing T1. In addition, we did not include data from participants who did not respond at T3 or T4. This resulted in a sample of 168, participants from which we imputed missing values. There were no missing values for the variables ECR anxiety and ECR avoidance. SPSS expectation maximization (EM) missing value analysis (MVA) were used to impute missing values for respondents with valid data for a minimum of 50% of the items of the PSI-CO variable, PSI-IS variable, PSI-SP variable, PSI-DP variable, PSI-RO variable, PSI-AT, PSI-HE, and the MPAS variable. Imputations were conducted for respondents participating in both T3 and T4. For the imputed variables, missing values were reduced by between 8.0 and 0.4%. EM was chosen as the manner of imputation, because PROCESS [42] was not able to analyze multiple imputed datasets.

## Results

### Descriptive statistics

Table 1 reports the demographic data. The mean age for the sample was 31.0 years. A large proportion of the participants reported wanting this pregnancy (95.8%), currently living with a partner (98.8%), having a gross annual household income above 751,000 NOK (85,125 USD; 70.8%), and having higher education (88.1%). Approximately half of the study sample were first-time mothers (49.4%). A substantial number of participants reported that they had previously felt depressed (32.7%) or were less able to enjoy the things they usually find enjoyable (40.5%) for a period of 2 weeks. Furthermore, 30.4% had been in contact with professionals for mental health issues at some point during their life. A small portion (3.6%) of mothers gave birth prematurely.

Table 2 reports the means, standard deviations, potential range and correlations for the study variables. The PSI-PD, PSI-PD5, and all included subscales of the PSI were significantly related to the ECR anxiety and avoidance. These correlations were positive and ranged from .24 (PSI-RO) to .52 (PSI-PD and PSI-PD5). The PSI-PD, PSI-PD5, and all included subscales of the PSI were significantly related to the MPAS. These correlations were negative and ranged from −.42 (PSI-SP) to −.67 (PSI-PD). The MPAS correlated significantly with ECR anxiety (−.40) and ECR avoidance (−.32).

**Table 2:** Descriptive statistics and Spearman’s correlations between study variables

	Mean	SD	Potential range	ECR Anxiety	ECR Avoidance	MPAS
ECR Anxiety	47.26	18.17	18–126			
ECR Avoidance	30.13	12.05	18–126	.48**		
MPAS	82.02	7.98	19–95	−.40**	−.32**	
PSI-PD	114.28	21.69	54–270	.52**	.42**	−.67**
PSI-PD5	89.87	18.41	42–210	.52**	.44**	**−.**65**
PSI-CO	23.07	5.49	13–65	.40**	.38**	−.56**
PSI-SP	16.61	4.26	7–35	.33**	.34**	−.42**
PSI-IS	12.55	3.83	6–30	.39**	.37**	−.45**
PSI-DP	17.07	5.42	9–45	.48**	.38**	−.54**
PSI-RO	20.58	4.38	7–35	.38**	.24*	−.54**

*ECR* Experiences in Close Relationships, *MPAS* Maternal Postnatal Attachment Scale, *PSI-PD* Parenting Stress Index – Parent Domain, *PSI-PD5* Parenting Stress Index – Parent Domain with 5 subscales, *CO* Competence, *SP* Spouse, *IS* Isolation, *DP* Depression, *RO* Role Restriction; *N* = 168; ^*^p < .01, ^**^p < .0001

### Hierarchical regression model with attachment style as a predictor of maternal infant bonding

Table 3 reports the hierarchical regression model that tested ECR anxiety and avoidance as predictors of scores on the MPAS. For the ECR subscales, variance inflation factors (anxiety 1.52 and avoidance 1.42) and tolerance (anxiety 0.66 and avoidance 0.70) did not indicate a serious problem with multicollinearity [43–45]. The first block with ECR anxiety and ECR avoidance was significant (*p* < .001), explaining 16% of the variance in the MPAS. In the second block, demographic information and allocation to the NBO or control group were added as predictors. The regression model was significant (*p* < .001), explaining 23% of the total variance in the MPAS. The third block that included the mental health-related variables was also significant (*p* < .001), explaining 25% of the total variance in the MPAS. The increase in explained variance between models one and two (*p* = .054) and models two and three (*p* = .162) was not significant. The ECR anxiety subscale was a significant individual predictor in the first two blocks (*p* < .05) but did not reach significance in the third block (*p* = .097). ECR avoidance was a significant predictor in all blocks (*p* < .05), with *p* = .009 in the third block.

**Table 3:** Hierarchical regression analysis testing ECR Anxiety and Avoidance as predictors of MPAS

Block	Predictors	*b* (95% CI)	SE B	*β*	*p*	R^2^
1	ECR Anxiety	−0.10 (− 0.18, − 0.03)	0.04	−.23	.005	.16
	ECR Avoidance	−0.16 (− 0.29, − 0.04)	0.06	−.23	.010	
2	ECR Anxiety	−0.08 (− 0.15, − 0.01)	0.04	−.18	.030	.23
	ECR Avoidance	−0.19 (− 0.34, − 0.07)	0.07	−.28	.005	
	NBO Group	0.01 (−2.35, 2.33)	1.19	.00	.992	
	Maternal age	0.07 (−0.23, 0.38)	0.16	.04	.664	
	Parenting experience	3.08 (0.61, 5.48)	1.25	.19	.014	
	Maternal education^a^					
	Upper secondary school or lower	3.52 (−1.01, 7.83)	2.25	.14	.110	
	Up to 4 years of higher education	1.30 (−1.20, 3.89)	1.30	.07	.327	
	Gross annual household income^b^					
	351,000–750,000 NOK (39,785–85,011 USD)	−4.95 (−11.40, 0.50)	3.01	−.27	.085	
	751,000 NOK (85,125 USD) or more	−3.90 (−9.99, 1.40)	2.88	−.22	.153	
3	ECR Anxiety	−0.07 (−0.14, 0.01)	0.04	−.15	.097	.25
	ECR Avoidance	−0.19 (− 0.33, − 0.06)	0.07	−.27	.009	
	NBO Group	0.04 (−2.28, 2.31)	1.16	.00	.967	
	Maternal age	0.15 (−0.16, 0.46)	0.16	.08	.358	
	Parenting experience	3.35 (0.89, 5.74)	1.25	.21	.009	
	Maternal education^a^					
	Upper secondary school or lower	3.98 (−0.66, 8.35)	2.30	.16	.078	
	Up to 4 years of higher education	1.49 (−1.07, 4.16)	1.33	.09	.266	
	Gross annual household income^b^					
	351,000–750,000 NOK (39,785–85,011 USD)	−4.87 (−12.12, 1.04)	3.33	−.26	.124	
	751,000 NOK (85,125 USD) or more	−4.42 (−11.31, 1.34)	3.20	−.25	.142	
	Mental health help seeking	0.86 (−2.28, 3.84)	1.57	.05	.588	
	Previous experience with being depressed	−3.02 (−6.59, 0.37)	1.79	−.18	.093	
	Previous lack of joy	−0.14 (− 3.23, 3.16)	1.62	−.01	.930	

*ECR* Experiences in Close Relationships, *NBO* Newborn Behavioral Observation; ^a^dummy coded variables with four or more years of higher education serve as a reference; ^b^dummy coded variables with 350,000 NOK (39,259 USD) or less serve as a reference; “Mental health help seeking” = having been in contact with professionals for mental health issues; “Previous experience with being depressed” = Previous experience with being depressed most of the day, almost every day for a period of 2 weeks; “Previous lack of joy” = Having previously had a 2-week period of diminished ability to enjoy things one has usually found enjoyable; standard errors and confidence intervals were based on 9992 percentile bootstrap samples as SPSS did not generate the requested 10,000 samples; *N* = 166

### Mediation analyses with parenting stress as a mediator

Mediation analyses testing PSI as a mediator between ECR and MPAS were performed in steps. First, we tested the full PSI-PD as a mediator. Then, we tested a reduced PSI-PD5 as a mediator. Finally, we tested the 5 subscales of the PSI-PD5 as mediators in the association between the ECR and MPAS. All potentially confounding variables from the last block of the hierarchical regression model were included as covariates in the mediation analyses. Confidence intervals for the direct and indirect effects were based on 10,000 bootstrap samples generated in the PROCESS. As there is no option for this in PROCESS, percentile bootstrap confidence intervals for the total effects were generated in separate regression analyses in SPSS. Approximately 10,000 bootstrap samples were generated for the total effects since the SPSS did not generate the requested 10,000 samples.

For all mediation models, there was a significant total effect of the ECR avoidance domain, *b* = − 0.230, 95% [− 0.360, − 0.107 CI], and the ECR anxiety domain, *b* = − 0.123, 95% CI [− 0.203, − 0.048], on the MPAS. There was no significant direct effect of the ECR avoidance domain on the MPAS when the PSI-PD, *b* = − 0.052, 95% CI [− 0.132, 0.033] or the PSI-PD5, *b* = − 0.063, 95% CI [− 0.150, 0.030], were included as a mediator. In the two separate mediation analyses, the ECR avoidance domain had significant indirect effects on the MPAS through PSI-PD, *b* = − 0.178, 95% CI [− 0.277, − 0.100] and PSI-PD 5, *b* = − 0.168, 95% CI [− 0.258, − 0.094]. There was also no significant direct effect of the ECR anxiety domain on the MPAS when the PSI-PD, *b* = − 0.006, 95% CI [− 0.055, 0.069], or the PSI-PD5, *b* = − 0.005, 95% CI [− 0.063, 0.054], were included as a mediator. In the two separate mediation analyses, the ECR anxiety domain had significant indirect effects on the MPAS through PSI-PD, *b* = − 0.128, 95% CI [− 0.188, − 0.080], and PSI-PD 5, *b* = − 0.118, 95% CI [− 0.176, − 0.073].

**Fig. 1:**
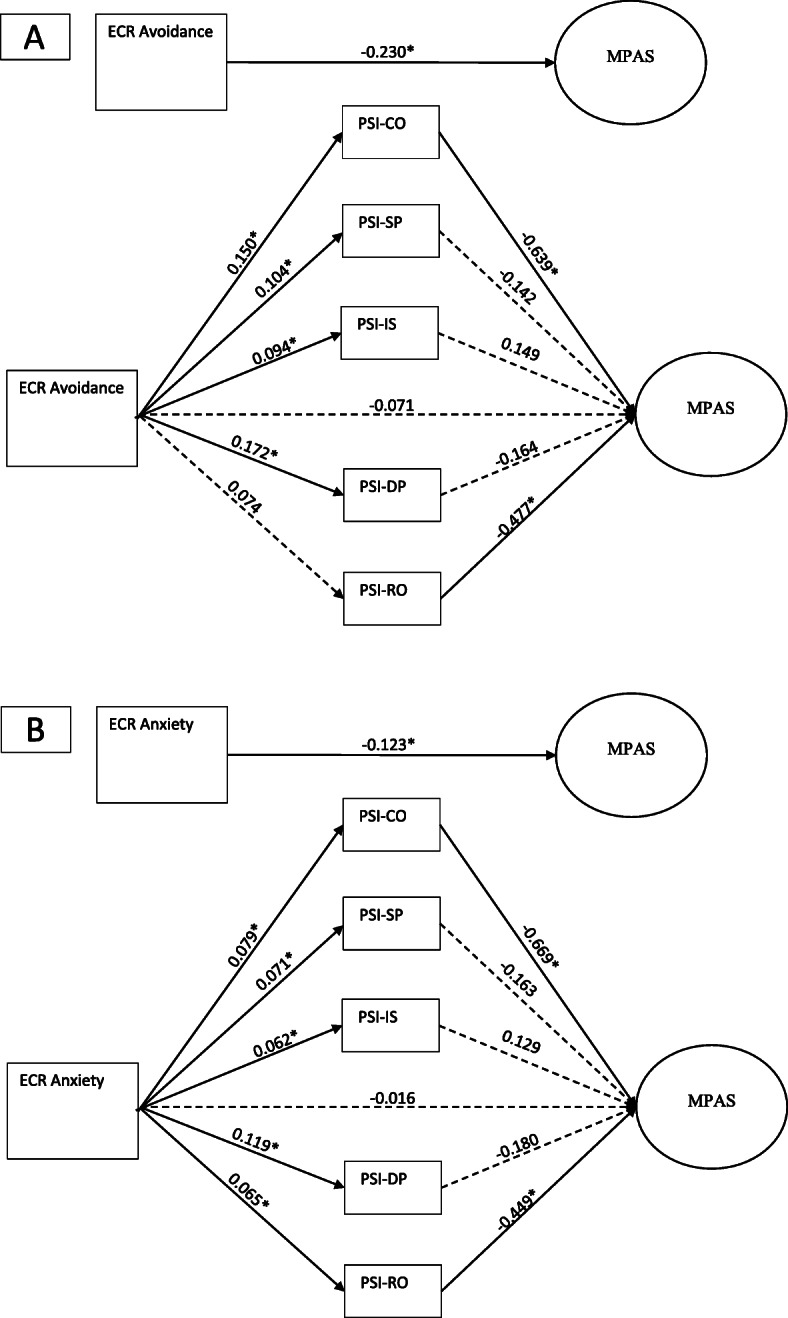
Mediation analyses testing the PSI subscales as mediators between the ECR subscales and the MPAS. *ECR* Experiences in Close Relationships, *MPAS* Maternal Postnatal Attachment Scale, *PSI* Parenting Stress Index, *CO* Competence, *SP* Spouse, *IS* Isolation, *DP* Depression, *RO* Role Restriction; The covariates from the hierarchical regression analysis are included in the mediation analyses: maternal age, parenting experience, education, gross annual household income, mental health help seeking, previous experience with being depressed, previous lack of joy, and NBO−/control group allocation; coefficients represent unstandardized coefficients; non-significant relationships are in dashed lines and significant relationships are in solid lines and are also highlighted with an asterix; significance level based on 95% percent CI from 10,000 bootstrap samples; *N* = 167

Figure 1 illustrates the two mediation analyses testing the 5 PSI subscales as mediators between the two ECR domains avoidance (Fig. 1a) and anxiety (Fig. 1b) and the MPAS. Regarding the ECR avoidance domain, there was no significant direct effect, *b* = − 0.071, 95% CI [− 0.152, 0.025], on the MPAS. The ECR avoidance had a significant indirect effect on the MPAS through the PSI CO, *b* = − 0.096, 95% CI [− 0.183, − 0.038]. See Fig. 1 for the estimates. Regarding the ECR anxiety domain, there was no significant direct effect, *b* = − 0.016 [− 0.077, 0.051] on the MPAS. The ECR anxiety had significant indirect effects on the MPAS through the PSI CO, *b* = − 0.053, 95% CI [− 0.107, − 0.018], and the PSI RO, *b* = − 0.029, 95% CI [− 0.066, − 0.005]. See Fig. 1 for the estimates.

## Discussion

The present study investigated the relationship between mothers’ adult attachment styles and mother-infant bonding, in addition to exploring the mediating effects of parenting stress. To the best of our knowledge, we are the first to explore parenting stress as a mediator between mothers’ attachment styles and mother-infant bonding. Our results show that a mother’s attachment style, especially an avoidant style, relates to poorer mother-infant bonding 8 weeks after birth. In addition, the relationship between both attachment dimensions and bonding was mediated by parenting stress. The stress variable sense of competence in the parenting role mediated the relationship between attachment avoidance and bonding, and sense of competence and role restriction mediated the relationship between attachment anxiety and bonding. Increased attachment avoidance and anxiety were related to increased stress, which was again related to decreased quality of bonding with the infant. Neither attachment-related avoidance nor attachment-related anxiety was directly related to mother-infant bonding when the parenting stress domains or subscales were included as mediators.

Our findings show that attachment avoidance and anxiety correlated negatively and significantly with maternal-infant bonding and positively with overall parenting stress and the stress subscales experience of competence in the parenting role, relationship with spouse, isolation, symptoms of depression and role restriction. This is in line with some previous studies that showed a relationship between adult attachment and different aspects of parenting [14], including parenting stress [32] and maternal-infant bonding [18], but contrary to one study that failed to find a significant association between adult attachment and maternal-infant bonding [16]. Further, in the hierarchical regression analysis, attachment anxiety and avoidance alone explained 16% of the variance in mother-infant bonding. Attachment anxiety did not reach significance in the last hierarchical regression model that included both adult attachment dimensions and that controlled for mental health history and demographic variables, but attachment anxiety was significant in the total effect of the mediation analysis when attachment avoidance was left out as a predictor. Attachment-related avoidance style addresses the degree to which one feels uncomfortable with closeness. In contrast, attachment-related anxiety concerns the extent of confidence one has in the availability and responsiveness of partners [12]. Normally, the mother-infant relationship is characterized by closeness, and the infant depends on his or her mother being psychologically available, interpreting his or her signals, giving comfort and taking care of his or her needs. Mothers high on the attachment avoidance dimension might feel uneasy and want to pull away from this new close relationship, thus impacting the quality of the mother-infant bond.

Separate mediation analyses for each adult attachment domain showed that the relationship with maternal-infant bonding was mediated by parenting stress, suggesting that parenting stress is important for the quality of maternal-infant bonding and that a mothers’s adult attachment style is associated with parenting stress in the postpartum period. Depression [17, 18] and childbirth-related posttraumatic stress disorder [18] have previously been suggested as mediators between the aforementioned variables, and our results suggest that parenting stress is a mediator that closely falls in line with these studies. Parenting stress was a significant mediator between adult attachment style and mother-infant bonding even when all confounding variables from the hierarchical regression analyses (e.g., demographics and mental health history) were included as covariates. Both versions of the domains of parenting stress were significant mediators in the relation between attachment dimensions and mother-infant bonding. For the included subscales of parenting stress, only mothers’ sense of competence in the parenting role was significant as a mediator between the avoidance attachment style and maternal-infant bonding. Competence concerns beliefs about the ability to take care of a child and make decisions as well as the ability to enjoy being a parent. For the anxiety attachment style, sense of competence and role restriction were significant as mediating variables in the relationship with maternal-infant bonding. Role restriction addresses the degree that a person experiences the parenting role as restricting, with a large focus on the child’s needs and limited room to care for oneself. For both dimensions of adult attachment, higher scores on these variables were related to increased parenting stress, which was again related to decreased quality of maternal-infant bonding. According to theory, attachment avoidance mirror the tendency to deactivate the attachment system and to avoid closeness with others [46]. Thus, one explanation for our finding may be that mothers with higher scores on attachment avoidance may, to a lower degree, experience the close relationship with their children as reinforcing. The same mediating path emerged for the anxiety attachment style. It could be that mothers with higher scores on this domain worry about not being competent enough to care for their baby and that this has a negative impact on the emotional bond with the baby. The mediating effect of role restriction between attachment anxiety and mother-infant bonding was surprising. Mothers high on attachment anxiety are thought to cling to their partners. A possible interpretation may be that becoming a mother might increase anxiety in mothers high on this dimension and thereby increase their feeling of being restricted in their new role. Further, having a baby might influence the family system by reducing the experience of closeness with one’s partner, and this may contribute to an experience of being restricted by the new baby and being less satisfied in the new role. Neither of the adult attachment dimensions showed a direct effect on maternal-infant bonding, meaning that the significant direct relationship between the attachment dimensions and bonding disappeared when parenting stress was taken into account. Mothers’ bond with their infants are under development in the postpartum period, and our results show that the stress that mothers experience, and thus how they experience handling this life transition, is an important factor in the bonding process.

### Implications

The results may have implications for research and clinical practice. There is a need for more studies on the relationship between adult attachment styles and maternal-infant bonding, including explorations of mental health-related variables as mediators. The association between mother-infant bonding and parenting stress is probably bidirectional, making the interpretation of our results difficult. Future studies should therefore also explore the mediating effect of parenting stress measured weeks before the assessment of mother-infant bonding to better grasp the direction of this relationship. In addition to conducting such mediation studies with adult attachment styles, this could also be investigated with attachment-related concepts such as mothers’ personality traits. Fathers are also important caregivers for children and should be included in future studies. Furthermore, future studies should also include a more disadvantaged sample than that included in the current study, as such a sample may have more problems in the areas of adult attachment style and mother-infant bonding and may reveal stronger results on the relationships between the study measures. In addition, a more at risk sample may reveal higher scores on the parenting stress variables than in the current study [47]. There is a possibility that variables such as stress related to isolation and support could have a higher impact as mediators in a more disadvantaged sample.

Exploring attachment-related mechanisms for the quality of mother-infant bonding may have clinical implications for the early identification of mothers at risk of having bonding difficulties. For example, as suggested by the results that the adult attachment style relates to parenting stress and that stress relates to bonding, reducing parenting stress in mothers of newborns with adult attachment difficulties may be an important intervention to increase mother-infant bonding. It may be beneficial to assess and aid in reducing parenting stress as early as before hospital discharge after childbirth in addition to follow up of highly stressed families after discharge. Parenting stress may have a range of sources as illustrated in this study. Thus, in addition to measuring an overall level of parenting stress, assessing subdomains of parenting stress might be of value as different profiles of parenting stress might benefit from different interventions. For example a mother high on stress related to sense of competence might find parental education and emotional support helpful, while high stress associated with the partner relationship will probably benefit from help in the direction of couple counseling (as suggested in [39]). Additionally, the effect of attachment based interventions for enhancing the quality of mother-infant bonding should be further investigated.

### Strengths and limitations

The main strengths of the present study are the longitudinal design and the use of well-established measures. In addition, participants answered comprehensive questionnaires on demographic information. The study also has some limitations. First, the sample mostly consists of well-educated, economically sound participants living with a partner. The sample was generally low on attachment anxiety and avoidance and reported mostly good quality of mother-infant bonding, which can indicate that the participants were generally well functioning. However, approximately one third of the participants reported experience of depression in the past, which might indicate some level of vulnerability. Second, parenting stress and mother-infant bonding were measured at the same time point. This means that even though the relationship between the variables was presented as stress mediating the relationship between attachment style and maternal-infant bonding, we cannot rule out a bidirectional relationship between these variables. Thus, the results of this study must be considered explorative. Exploring parenting stress as a mediator seems theoretically reasonable, and some argue that this justifies the use of mediation analyses of cross-sectional data [42]. Future studies should further explore the causal relationship between these variables. Third, the regression models are a clear simplification of reality, and the inclusion of other potentially relevant variables (e.g., infant temperament and mothers’ temperament) might have changed our results. Fourth, all data were collected using self-reported measures. There is a possibility that an interview-based perspective on adult attachment might have given different results. In addition, assessing the parent-infant relationship with observational measures might have also given more information. Fifth, a larger sample size would have been preferable, given the large numbers of predictors in the regression analyses. Sixth, participants answered the measurement points at a span in weeks of gestation and weeks after birth. For exemple were the range of responding to T4 from 5 to 15 weeks after birth. The mother-infant bond may be different at 5 weeks after birth compared with 15 weeks after birth, and this might have impacted the results of the study.

## Conclusion

Our results show that a mother’s adult attachment style relates to her bonding with her infant. The avoidant adult attachment style was a significant individual predictor of maternal-infant bonding even when including a number of relevant confounding variables. Mediation analyses revealed that parenting stress is a meditator in this relationship. The overall parenting stress domain and the subscale of competence in the parenting role mediated the relationship between adult attachment avoidance and bonding. The overall parenting stress domain and the subscales of competence and role restriction mediated between attachment anxiety and bonding. The results may have implications for the early identification of mothers at risk of having bonding difficulties. We encourage more studies on attachment-based mechanisms for the quality of mother-infant bonding and on disentangling the relationships between attachment style, parenting stress and mother-infant boding.

## Data Availability

The dataset used during the study is available from the corresponding author on request.

## Abbreviations

ECR: Experiences in Close Relationships
NBO: Newborn Behavioral Observation
MPAS: Maternal Postnatal Attachment Scale
PSI-PD: Parenting Stress Index-Parent Domain
PSI-PD5: Parenting Stress Index-Parent Domain with 5 subscales
PSI-AT: Parenting Stress Index-Attachment
PSI-CO: Parenting Stress Index-Competence
PSI-DP: Parenting Stress Index-Depression
PSI-HE: Parenting Stress Index-Health
PSI-IS: Parenting Stress Index-Isolation
PSI-RO: Parenting Stress Index-Role Restriction
PSI-SP: Parenting Stress Index-Spouse
